# Cardio- and cerebrovascular responses to the energy drink *Red Bull* in young adults: a randomized cross-over study

**DOI:** 10.1007/s00394-014-0661-8

**Published:** 2014-01-29

**Authors:** Erik K. Grasser, Gayathri Yepuri, Abdul G. Dulloo, Jean-Pierre Montani

**Affiliations:** Laboratory of Integrative Cardiovascular and Metabolic Physiology, Division of Physiology, Department of Medicine, University of Fribourg, Chemin du Musée 5, 1700 Fribourg, Switzerland

**Keywords:** Energy drink, Blood pressure, Cerebral blood flow velocity, Microvascular endothelial dysfunction, Hemodynamics, Risk factor

## Abstract

**Purpose:**

Energy drinks are beverages containing vasoactive metabolites, usually a combination of caffeine, taurine, glucuronolactone and sugars. There are concerns about the safety of energy drinks with some countries banning their sales. We determined the acute effects of a popular energy drink, *Red Bull,* on cardiovascular and hemodynamic variables, cerebrovascular parameters and microvascular endothelial function.

**Methods:**

Twenty-five young non-obese and healthy subjects attended two experimental sessions on separate days according to a randomized crossover study design. During each session, primary measurements included beat-to-beat blood pressure measurements, impedance cardiography and transcranial Doppler measurements for at least 20 min baseline and for 2 h following the ingestion of either 355 mL of the energy drink or 355 mL of tap water; the endothelial function test was performed before and two hours after either drink.

**Results:**

Unlike the water control load, *Red Bull* consumption led to increases in both systolic and diastolic blood pressure (*p* < 0.005), associated with increased heart rate and cardiac output (*p* < 0.05), with no significant changes in total peripheral resistance and without diminished endothelial response to acetylcholine; consequently, double product (reflecting myocardial load) was increased (*p* < 0.005). *Red Bull* consumption also led to increases in cerebrovascular resistance and breathing frequency (*p* < 0.005), as well as to decreases in cerebral blood flow velocity (*p* < 0.005) and end-tidal carbon dioxide (*p* < 0.005).

**Conclusion:**

Our results show an overall negative hemodynamic profile in response to ingestion of the energy drink *Red Bull*, in particular an elevated blood pressure and double product and a lower cerebral blood flow velocity.

## Introduction

Energy drinks refer to a category of sugary drinks that also include variable amounts of caffeine, taurine and glucuronolactone as well as other ingredients that may include vitamins and minerals [1]. Their popularity has substantially increased since their introduction around 1960 [2], and energy drinks are now one of the fastest growing segments in the beverage industry [3]. Today, the majority of energy drinks are targeted toward adolescents and young adults [4], and the manufacturer’s publicity claims positive effects on overall performance, mental concentration, reaction speed, vigilance, metabolism and well-being if such a beverage is consumed [5]. Despite these claims for beneficial effects, there are health concerns about these energy drinks because of reported side effects like cardiovascular complications or intoxication symptoms [6–8].

There is, however, little robust scientific investigation about the potential health risks associated with energy drinks. Studies investigating the direct impact of energy drinks on the cardiovascular system are few, and the results are not always coherent. A decade ago, Baum and Weiss [9] investigated the impact of the energy drink *Red Bull* (RB) on cardiac parameters before and after exercise in trained athletes and found that ingestion of 500 mL of RB did not lead to significant changes in heart rate or stroke volume when assessed within 40 min post-drink under resting conditions; however, increased left atrium contractility leading to increased stroke volume was observed later during the post-exercise recovery period. A few months later, Alford et al. [10] investigated the effects of RB on exercise performance and mood in two separate experiments, and reported that consumption of 250 mL of RB did not alter resting blood pressure (BP) 30 min later. Similarly, Bichler et al. [11] reported no change in BP nor in heart rate within 45 min after ingesting capsules containing caffeine and taurine in amounts equivalent to those found in a 250 mL RB drink. More recently, Worthley et al. [12] compared 250 mL of a sugar-free energy drink of similar composition as RB versus a water control and found an increase in BP without a change in heart rate at one hour post-drink. By contrast, Ragsdale et al. [13], who compared 250 mL of RB (normal calorie and low calorie) with a control drink, found no changes in overall cardiovascular function as measured by BP and heart rate throughout a 2-h test period. Furthermore, while Nienhueser et al. [14] reported that 473 mL of RB had no significant effect on heart rate over an one hour post-drink period, Steinke et al. [15] reported that consumption of 500 mL of an energy drink of unspecified brand, but containing caffeine and taurine in amounts equivalent to two 250 mL cans of RB, resulted in significant increases in heart rate as well as in BP at 2 and 4 h post-drink.

Some of the discrepancies across these above-mentioned studies could be attributed to differential diet and lifestyle of the test subjects, state of fitness (trained vs. untrained), posture during the experiment, the use of different energy drinks varying in amount and type of active metabolites and different volume loads of the same type of energy drink under investigation. Furthermore, several of these studies were not specifically designed to study postprandial cardiovascular responses as judged by the lack of tight control of food and beverage consumption on the study day before the test, too short duration of post-drink monitoring, the lack of an appropriate control drink in some studies, as well as by the limitations for detecting small-to-modest changes in BP due to its infrequent measurements during baseline and post-drink periods. In fact, no study utilized continuous beat-to-beat hemodynamics measurements. Furthermore, despite the controversy about a possible mental effect of energy drinks and the abundance of publications on this topic, no study used a transcranial Doppler approach to evaluate cerebral blood flow velocity.

The objective of the study reported here was to investigate the acute cardiovascular and cerebrovascular responses to the popular energy drink RB under standardized experimental pre-drink and post-drink conditions, and with the use of state-of-the-art techniques for a continuous and comprehensive monitoring of cardiovascular and cerebrovascular functions. We determined, in a randomized crossover study, the beat-to-beat cardiovascular and cerebrovascular responses to oral ingestion of RB and tested the hypothesis that ingestion of this energy drink will impact upon the cardiovascular system leading to increased BP and double product. Since the endothelium plays an important role for the function of resistance vessels, we also tested the hypothesis that microvascular endothelial dysfunction would be a cause of elevated BP in response to the energy drink.

## Methods

### Subjects

Twenty-five healthy young adults (12 women, 13 men) aged 20–31 years (mean 22.5 ± 0.6) were recruited from our local University student population and their friends. The mean height of the participants was 173 ± 2 cm, body weight 70 ± 2 kg and their body mass index (BMI) was 23.3 ± 0.6 kg m^−2^. Exclusion criteria included those with a BMI greater than 30 kg m^−2^, competition athletes and individuals with a daily exercise workload exceeding 60 min per day. None of the subjects had any diseases or were taking any medication affecting cardiovascular or autonomic regulation and none reported caffeine intake in excess of 150 mg daily from food and beverages. Based on a questionnaire, 15 subjects (6 vs. 9) were low caffeine users with an estimated daily intake of approximately 60 mg, while 10 subjects (6 vs. 4) were caffeine naïve. The questionnaire included coffee and energy drink consumption. All participants were studied in the morning after an overnight (12 h) fast, and they were requested to avoid alcohol or caffeine for at least 24 h prior to the test. Written informed consent was obtained from each test subject. The study protocol complied with the Declaration of Helsinki and received local ethics committee approval.

### Study design

All experiments took place in a quiet, temperature-controlled (20–22 °C) laboratory and started between 08.00 and 09.00 a.m. Every subject attended two separate experimental sessions (each session separated at least by 2 days) according to a randomized crossover study. Randomization was performed using a random sequence generator (http://www.random.org/sequences/) where the session order was determined for 25 test subjects before the study started (1 = Red Bull, 2 = Water). Test subjects were not allowed to know the order of their sessions until they had their first drink. On arrival at the laboratory, subjects were asked to empty their bladders if necessary and to sit in a comfortable armchair. The cardiovascular monitoring equipment was then connected. Following a variable period for reaching cardiovascular and metabolic stability (at least 30 min), the microvascular function test was performed (time required: about 30 min). A baseline recording was then made for 20 min, starting with the beat-to-beat measurements. Then, the test subjects ingested non-blinded either 355 mL of a degased energy drink RB containing caffeine (114 mg), taurine (1,420 mg), glucuronolactone (84.2 mg), sucrose and glucose (39.1 g) or 355 mL of tap water at room temperature. Subjects were asked to ingest their drink in a convenient pace over 4 min. After 2 h of post-drink cardiovascular recording, the microvascular function test was repeated. Throughout the procedures, subjects were permitted to watch neutral documentaries on a flat TV screen set at eye level.

### Cardiovascular recordings

Cardiovascular recordings were performed using a *Task Force Monitor* (TFM) (*CNSystems*, Medizintechnik, Graz, Austria) with data sampled at a rate of 1,000 Hz [16]. Continuous BP was monitored using the Penaz principle from either the index or middle finger of the right hand and was calibrated to oscillometric brachial BP measurements on the contralateral arm. Impedance cardiography measurements [17–19], in which the changes in thoracic impedance are converted to reflect changes in thoracic fluid content/volume over time, were performed based on the original *Kubicek* [20, 21] approach but using an improved estimate of thoracic volume [22], which allows calculation of cardiac stroke volume. ECG/Impedance electrodes were positioned together with upper arm and finger BP cuffs. Electrode strips were placed at the neck and thoracic regions, the latter specifically at the midclavicular at the xiphoid process level (*CNSystems* standard electrode kits).

### Transcranial Doppler measurements

Cerebral blood flow velocity was measured using transcranial Doppler ultrasonography (*Doppler*-*Box*, DWL, Sipplingen, Germany). The left and right middle cerebral artery was insonated at a depth of 40–55 mm using a 2-MHz probe, which was fixed in place with an adjustable headset. Beat-to-beat values of systolic, diastolic and mean velocity were recorded and merged real-time with the TFM. Expiratory air was sampled via a nasal cannula, and end-tidal CO_2_ measured by infrared absorption (*Datex*, Multicap, Instrumentarium Corp., Helsinki, Finland).

### Microvascular endothelial function

Microvascular endothelial function was assessed non-invasively in the finger skin microcirculation by a combination of iontophoresis and laser Doppler flowmetry (*Perimed* PF5010, Stockholm, Sweden), using a standard protocol [23] which is briefly described as follows: acetylcholine (1 %, *Fluka*, Sigma-Aldrich Chemie GmbH, Steinheim, Germany) was delivered to the middle dorsal phalanx of the third finger of the non-dominant hand using an anodal electrical current (0.1 mA for 20 s) and consisted of seven doses of acetylcholine with a 60-s interval between each dose. The electrical current was conveyed by a battery power supply that was isolated from the mains electricity. Then, sodium nitroprusside (0.01 %, *Riedel*–*de Haen*, Sigma-Aldrich Laborchemikalien GmbH, Seelze, Germany) was delivered to the same spot of the fourth finger using a cathodal current (0.2 mA for 20 s) and consisted of nine doses of sodium nitroprusside with a 90-s interval between each dose. The skin perfusion responses were recorded by a laser Doppler flowmetry probe, and the probes temperature was kept constantly at 32 °C during all measurements. Coefficient of variation of pre-drink baseline values to acetylcholine was 39 ± 7 % and for sodium nitroprusside 33 ± 7 %, determined from all pre-drink tests, which is in agreement with a previous study [24].

### Data analysis

Values of cardiac RR interval, systolic BP (SBP) diastolic BP (DBP), cerebral blood flow velocity (CBFV), end-tidal CO_2_ (etCO_2_) and breathing frequency (BF) were averaged every 10 min during baseline and every 20 min during the 2 h post-drink period. Heart rate (HR) was calculated from the appropriate RR-Interval. Cardiac output (CO) was computed as the product of stroke volume (SV) and HR. Mean arterial blood pressure (MAP) was calculated from DBP and SBP as follows: MAP = DBP + 1/3 (SBP–DBP). Total peripheral resistance (TPR) was calculated as MAP/CO. Double (rate pressure) product (DP) was calculated as HR x SBP and provides valuable information for the oxygen consumption of the myocardium [25]. Cerebrovascular resistance (CVRI) was calculated as the mean blood pressure at brain level (BP_mean_brain_) divided by CBFV_mean_. We estimated BP_mean_brain_ as the difference between BP_mean_ (MAP) at heart level and the hydrostatic pressure (BP_hydro_) effect at the level of transcranial insonation. We determined the vertical length (*h*) between the insonation site and the fourth intercostal space in the midclavicular line (heart level). The hydrostatic pressure of the blood column between heart and insonation levels was calculated as BP_hydro_ = *ρ* **×** *g* **×** *h*, where *ρ* is the specific density of blood (1.06 g/cm^3^) and *g* is the gravitational acceleration (9.81 m/s^2^). Acetylcholine- and sodium nitroprusside-mediated vasodilation were calculated as the absolute increase in arbitrary units from baseline to the average of the final two deliveries.

### Statistical analysis

All values are reported as mean ± SE. Statistical analysis was performed by two-way *ANOVA* for repeated measures with time and treatment (drink type) as within-subject factors using statistical software (*Statistix* version 8.0, Analytical Software, Tallahassee, FL 32317, USA). Where significant differences were found, the effects of each drink over time were analyzed by comparing values at each time-point over the post-drink period with the basal values recorded during the 20 min immediately before drinking using one-way *ANOVA* with *Dunnett’s* multiple comparison test or the *Friedman* test with *Dunn’s* post hoc testing. Variables were tested for normality using the *D’Agostino & Pearson* omnibus normality test. A paired *t* *test* or *Wilcoxon* matched pairs test was used to compare the post-drink effect between the drinks. A *Friedman* test with *Dunn’s* multiple comparison post hoc analysis was used to compare vasodilatory responses before and after drug administration (all performed with *GraphPad Prism*, Version 5, San Diego, CA, USA). All reported *p* values are two-sided. For all tests, significance was set at *p* ≤ 0.05.

## Results

### Subject characteristics

The test subject characteristics are presented in Table 1. Baseline pre-drink values on both test days were similar for hemodynamic, transcranial and microvascular measurement parameters. No subject reported gastrointestinal symptoms or other unpleasant effects after ingestion of the drinks.

**Table 1:** Baseline hemodynamic and transcranial Doppler data recorded 20 min before ingesting both drinks and baseline laser Doppler perfusion for microvascular measurements

	Energy drink	Water
Systolic blood pressure (mmHg)	114 ± 2	113 ± 2
Mean blood pressure (mmHg)	87 ± 1	86 ± 1
Diastolic blood pressure (mmHg)	73 ± 1	73 ± 1
Heart rate (beats min^−1^)	59 ± 2	60 ± 1
Double product (mmHg beats min^−1^)	6,742 ± 197	6,740 ± 160
Stroke volume (mL)	83 ± 2	83 ± 2
Cardiac output (L min^−1^)	4.83 ± 0.11	4.93 ± 0.12
Total peripheral resistance (mmHg min L^−1^)	18.1 ± 0.5	17.8 ± 0.5
Cerebral blood flow velocity (cm s^−1^)	65 ± 4	61 ± 3
Cerebrovascular resistance (mmHg s cm^−1^)	1.46 ± 0.09	1.48 ± 0.09
End-tidal carbon dioxide (mmHg)	36.5 ± 0.4	35.9 ± 0.5
Acetylcholine^a^ (AU^b^)	27.8 ± 3.4	25.9 ± 3.3
Sodium Nitroprusside^a^ (AU^b^)	20.2 ± 2.3	21.2 ± 3.2

Data are presented as mean ± SE; *n* = 25 (12 women, 13 men)

^a^Pre-drink skin blood flux related baseline values, average over 30 s

^b^AU means arbitrary units

### Cardiovascular responses

**Fig. 1:**
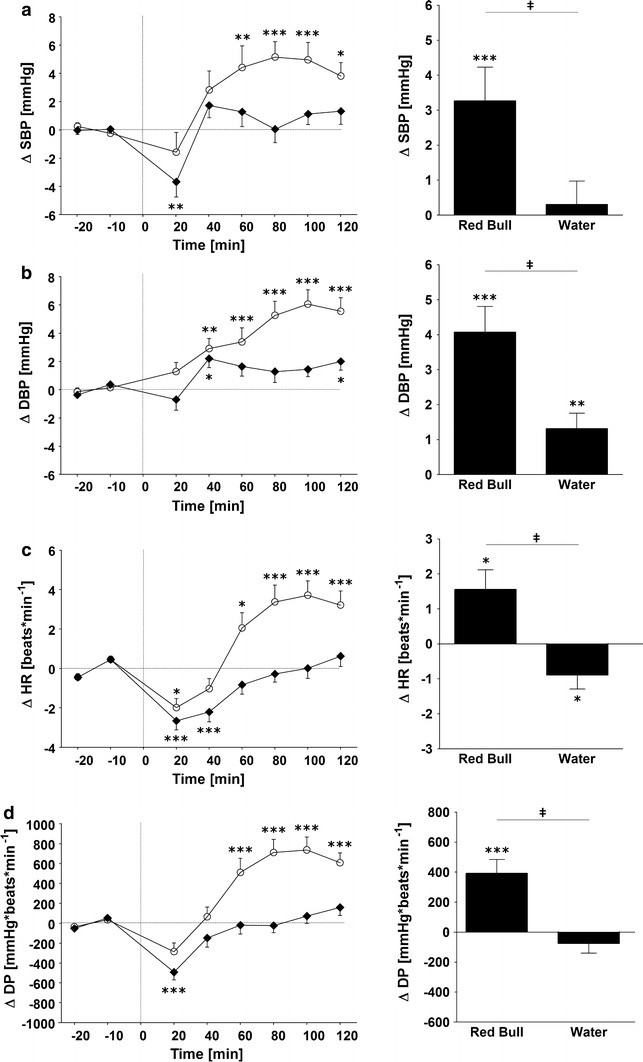
*Left panel* Time course of changes in systolic blood pressure (SBP) (**a**), diastolic blood pressure (DBP) (**b**), heart rate (HR) (**c**) and double product (DP) (**d**) before and after ingestion of *Red Bull* (*open circle*) and water (*solid rhombus*). *Right panel* Average changes over 120 min post-drink, equivalent to area under the curve. *p < 0.05, **p < 0.01 and ***p < 0.005, statistically significant differences over time from baseline values (*left* and *right* *panel*). **‡** *p* < 0.005, statistically significant differences between responses to the drinks (*right panel*). *Time 0* indicates resumption of the recordings after the 4-min drink period

Changes for SBP, DBP, HR and DP are presented in Fig. 1. Compared to baseline values, RB ingestion led to increases both in SBP and DBP as from 20 min post-drink, with the SBP peak (5.2 ± 1.0 mmHg, around 70 min) being reached earlier compared to the DBP peak (6.1 ± 1.1 mmHg, around 90 min). The BP-elevating effects of RB are also found when compared to the water load, with the effect of the RB drink resulting in significantly higher values for SBP (3.3 ± 1.0 vs. 0.3 ± 0.7 mmHg, *p* < 0.005) and DBP (4.1 ± 0.7 vs. 1.3 ± 0.4 mmHg, *p* < 0.005) if values were averaged over 120 min post-drink. Ingestion of either water or RB led to a drop in HR below baseline values over the first 40 min with the water load effect being significant. Afterwards, HR after RB ingestion rose steadily above baseline or relative to water load values reaching a peak around 90 min (3.7 ± 0.7 beats min^−1^), followed by a subsequent decreasing trend. Ingestion of RB also significantly increased the double product (DP) (391 ± 94 vs. −75 ± 65 mmHg beats min^−1^, *p* < 0.005) compared to water, with a peak around 90 min (737 ± 130 mmHg beats min^−1^).

**Fig. 2:**
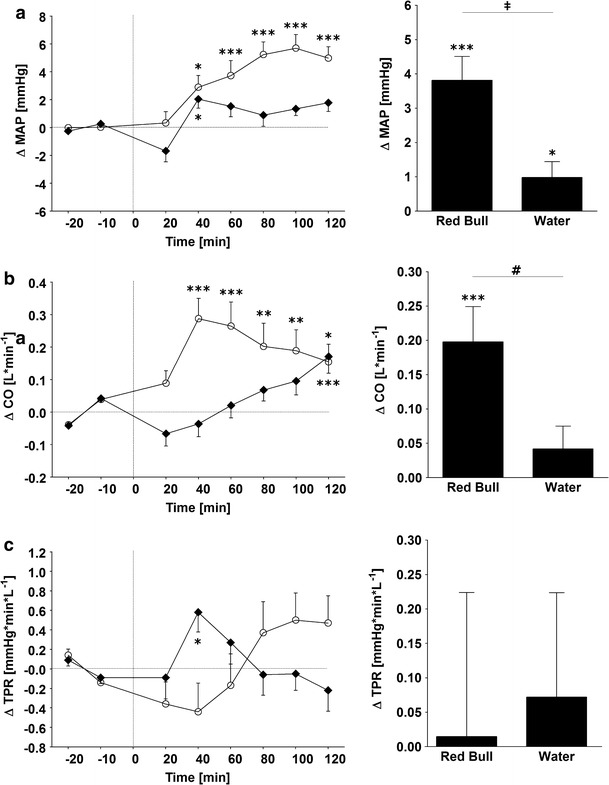
*Left panel* Time course of changes in mean arterial blood pressure (MAP) (**a**), cardiac output (CO) (**b**) and total peripheral resistance (TPR) (**c**), following the ingestion of *Red Bull* (*open circle*) or water control (*solid rhombus*). *Right panel* Average changes over 120 min post-drink, equivalent to area under the curve. *p < 0.05, **p < 0.01 and ***p < 0.005, statistically significant differences over time from baseline values. **‡** *p* < 0.005, **#** *p* < 0.05 statistically significant differences between responses to the drinks (*right panel*). *Time 0* indicates resumption of the recordings after the 4-min drink period

Changes for MAP, CO and TPR are presented in Fig. 2. In response to the RB drink, the MAP slowly started to increase over baseline values around 30 min post-drink, reaching its peak at a time-point which is later than for CO, namely at about 90 min (5.7 ± 1.0 mmHg). On the other hand, CO immediately rose over baseline values, peaking around 30 min (0.28 ± 0.06 L* min^−1^). When the MAP and CO responses to RB drink are compared to those of the water load, they are significantly higher with RB, namely 3.8 ± 0.7 vs. 1.0 ± 0.5 mmHg (*p* < 0.005) for MAP and 0.20 ± 0.05 vs. 0.04 ± 0.03 L min^−1^ (*p* < 0.05) for CO. Calculations of total peripheral resistance from BP and CO indicate no significant changes with RB relative to baseline values or to the water load (Fig. 2).

### Endothelial function

**Fig. 3:**
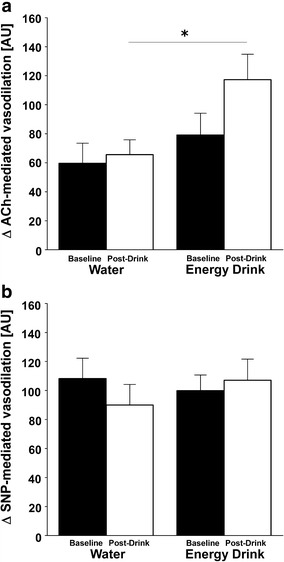
Microvascular measurements before and 2 h after the drink. ACh (**a**) (acetylcholine) and SNP (**b**) (sodium nitroprusside). Baseline refers to the average of the last two applications either of ACh or SNP 20 min prior either drink. Post-drink refers to the average of the last two applications either of ACh or SNP 2 h after either drink * *p* < 0.05, statistically significant difference between post-drink conditions. *AU* arbitrary units

No significant differences are observed in baseline values for microvascular endothelial flux when assessed on either day prior to ingestion of RB or water (Table 1). In comparison with the response to the water load, however, RB ingestion resulted in a significant increase in the response to acetylcholine-mediated vasodilation (66 ± 10 vs. 117 ± 18 AU, *p* < 0.05), but did not influence sodium nitroprusside-mediated vasodilation (Fig. 3).

### Cerebrovascular responses

**Fig. 4:**
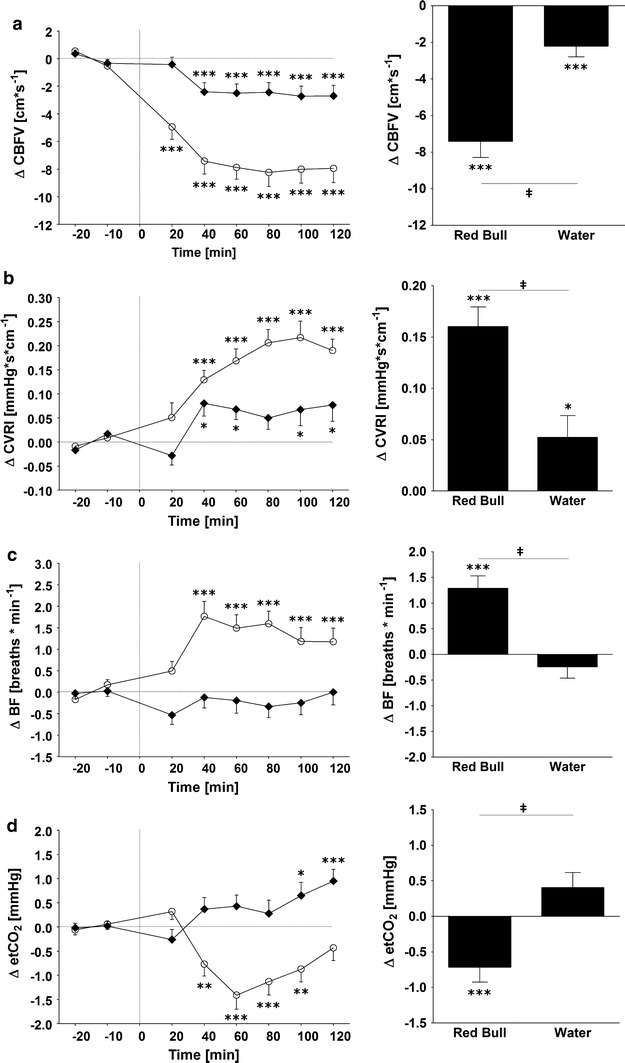
*Left panel* Time course of changes in cerebral blood flow velocity (CBFV) (**a**), cerebrovascular resistance (CVRI) (**b**), breathing frequency (BF) (**c**) and end-tidal carbon dioxide (etCO_2_) (**d**) following ingestion of Red Bull (*open circle*) and water (*solid rhombus*). *Right panel* Average changes over 120 min post-drink, equivalent to area under the curve. *p < 0.05, **p < 0.01 and ***p < 0.005, statistically significant differences over time from baseline values. **‡** *P* < 0.005, statistically significant differences between responses to the drinks (*right panel*). *Time 0* indicates resumption of the recordings after the 4-min drink period

Figure 4 shows the changes over time for CBFV, CVRI, BF and etCO_2_. Immediately after ingestion of RB, the CBFV started to decline with a negative peak (−8.2 ± 1.0 cm s^−1^) around 70 min, while CVRI rose gradually above baseline levels, peaking around 90 min (0.22 ± 0.03 mmHg s cm^−1^). Ingestion of water also decreased CBFV and increased CVRI significantly over time but the effect is far less pronounced compared to RB. This is reflected in a significant difference between RB and water if values were averaged over 120 min post-drink for CBFV (−7.4 ± 0.9 vs. −2.2 ± 0.6 cm s^−1^, *p* < 0.005) and CVRI (0.16 ± 0.02 vs. 0.05 ± 0.02 mmHg s cm^−1^, *p* < 0.005). The data on changes in BF and etCO_2_ show that after an initial stable period for 20 min, etCO_2_ started to decline and BF to increase in response to RB (but not with water), with a peak for etCO_2_ around 50 min (−1.4 ± 0.3 mmHg) and for BF around 30 min (1.8 ± 0.4 breaths min^−1^). Subsequently, whereas etCO_2_ in response to RB returned slowly toward the baseline levels, BF remained elevated above baseline levels even at the end of the test, i.e., at 120 min post-drink. Analyses of the average values over the entire post-drink study time indicate significant differences with RB compared to water both for etCO_2_ (−0.7 ± 0.2 vs. 0.4 ± 0.2 mmHg, *p* < 0.005) and BF (1.28 ± 0.25 vs. −0.24 ± 0.23 breaths min^−1^, *p* < 0.005).

## Discussion

The aim of this study was to evaluate the acute cardio- and cerebrovascular changes in response to a popular commercially available energy drink, RB. To the best of our knowledge, this is the first study that has evaluated the influence of an energy drink using beat-to-beat hemodynamics and cerebrovascular measurements. We show that ingestion of one can of RB resulted in an augmented workload to the heart as evidenced by elevated BP, HR, CO and DP values. Based on our findings that ingestion of RB does not lead to diminished microvascular endothelial function in response to acetylcholine, our study suggests that impaired endothelial function, at least in the microvasculature, is unlikely to account for the increased BP-elevating effect of RB. Furthermore, consumption of RB substantially decreased cerebral blood flow velocity and increased cerebrovascular resistance, which stands in agreement with the observed reduction in etCO_2_. The observed overall negative hemodynamic profile in response to one can of an energy drink could aggravate pre-existing health problems and warrants further studies using appropriate patient groups.

Energy drinks are one of the most rapidly increasing beverages promoted aggressively for their claimed beneficial effects on body and mental strength, but the data presented here suggest that consumption of energy drinks is potentially harmful because of the extra cardiac work load and the decreased cerebral blood flow velocity observed during resting conditions. While our data here showing BP-elevating effects of RB in young adults appear to be in conflict with several past studies in which RB ingestion was not found to increase BP, two main explanations can be put forward to account for these apparent discrepancies.

First, analysis of the time course of the changes in BP and heart rate in our study indicates that differences in response to the RB drink versus water control only became statistically significant as from 1 h post-drink, with peak values being reached between 80 and 90 min. Our study thus underscores the potential importance of assessing the cardiovascular effects of RB for periods lasting at least 1 h. This contention is consistent with the results of Alford et al. [10] and those of Baum and Weiss [9] where no BP-elevating effect of 250 or 500 mL of RB, respectively, was observed at 30 or 40 min post-drink. It is also in agreement with the data of Bichler et al. [11] where a change in BP or heart rate could not be demonstrated within 45 min after ingesting capsules containing 100 mg of caffeine and 1,000 mg of taurine, i.e., in amounts equivalent to those found in a 250 mL RB drink. Conversely, our data showing that BP-elevating effects of RB became significant between 1 and 2 h post-drink was in line with the findings of Worthley et al. [12] where an increase in BP was found 1 h after ingesting 250 mL of a sugar-free RB-like drink compared to a lack of change after water ingestion. Our data are also consistent with the report of Steinke et al. [15] that consumption of 500 mL of an energy drink that is similar in its composition to RB resulted in significant increases in heart rate (+5–7 beats per min) as well as in systolic and diastolic BP (+4–8 mmHg) between 1 and 4 h post-drink. Thus, unless the assessment of cardiovascular responses to RB and other energy drinks are conducted over periods of 1 h or more, there is a high risk for false-negative results.

Second, differences in the acute cardiovascular responses to RB between our study showing increased BP compared to those showing no effect can also be related to the use of different methodological approaches for measuring BP. Unlike our approach that measured BP by continuous beat-by-beat hemodynamics monitoring, the measurement of BP in previously reported studies by sphygmomanometry only occasionally throughout the experiment is likely to lack the degree of sensitivity required to detect statistically significant modest changes in BP. For example, Ragsdale et al. [13], in a double-blind experiment in 68 participants where 250 mL of RB was compared to control drinks, reported no changes in BP over a 2 h post-drink period with BP assessed by sphygmomanometry only at 0, 60 and 120 min in response to RB. A more detailed analysis of their data, however, reveals that at the 60 min measurement time-point, BP had increased by 3 mmHg (but non-significantly) in response to the RB drink but not with the control drink. In light of our findings that both systolic and diastolic BP peaks at 80–90 min post-drink, one therefore cannot disregard the possibility that this tendency of an increase in BP, with the RB at 60 min post-drink in Ragsdale’s study [13], may have been detected as a significant increase by continuous measurement of BP over the 2-h test period.

We conducted microvascular endothelial function testing to investigate whether hemodynamic changes following ingestion of energy drinks are linked to endothelial dysfunction. Using peripheral arterial tonometry to investigate a potential role of energy drinks in endothelial dysfunction, Worthley et al. [12] presented detrimental effects on endothelial function in their study. In addition, a case report revealed abnormal endothelial function, worse at 90 min following ingestion of a 24 oz (710 mL) *Monster* energy beverage using the brachial flow-mediated dilation method [26]. These appear to be in contrast to our findings where acetylcholine-mediated endothelial function showed an augmented vasodilation after consumption of RB. Explanations for these differential findings may be related to differences in the method utilized to investigate endothelial function (i.e., microvascular endothelial function testing, which uses iontophoresis with acetylcholine and sodium-nitroprusside vs. flow-mediated dilation, which assesses the diameter of the brachial artery in response to reactive hyperemia using an ultrasound technique), differences in sugar content, the total volume of drink consumed and to differences in the content of caffeine and taurine in the drinks utilized. Our subjects ingested 355 mL RB containing 39.1 g of sugar while Worthley et al. [12] used a sugar-free energy drink with 250 mL drink volume containing also less caffeine and taurine compared to the RB drink in our study. Furthermore, a recent publication focusing on the impact of acute administration of caffeine on vascular function found that caffeine augments endothelium-dependent vasodilation in a healthy young subpopulation [27]. However, caffeine may reduce myocardial blood flow during exercise, and therefore, given that many consume energy drinks and then exercise, this is an area that needs further study [28]. Furthermore, as somnolence due to sleep deprivation is often an underlying reason for young people to consume energy drinks, studies investigating the interaction between caffeinated beverages and sleep deprivation on vascular functions are also warranted. On the other hand, it was observed that a daily taurine supplementation of 1.5 g in healthy humans had a beneficial impact on microvascular endothelial function in smokers as well as in control non-smokers [29]. Because our study’s focus was on the cardiovascular responses to the energy drink *per se* rather to its specific ingredients, we can only conclude that microvascular endothelial dysfunction is not responsible for our observed increase in BP in response to RB. This conclusion is further supported through our hemodynamic beat-to-beat derived data where no change in the total peripheral resistance could be observed.

To our knowledge, this is the first documentation where cerebral blood flow velocity in response to ingestion of an energy drink has been evaluated. In our study, cerebral blood flow velocity started to decline immediately after the drink, reaching a minimum at 80 min and remained below baseline levels for at least 120 min post-drink. This was accompanied by an increased cerebrovascular resistance which could in part account for the observed decrease in velocity. As CO_2_ is known as one of the strongest metabolites affecting cerebral blood flow [30, 31], our findings that etCO_2_ levels are significantly decreased in response to the RB drink suggest that the observed drop in cerebral blood flow velocity and the accompanied rise in cerebrovascular resistance could be due, at least partly, to the change in etCO_2_ levels. Indeed, when taken together with the results of a previous study [32] which in evaluating the role of oral administered caffeine on cerebral circulation could not find a relation between CO_2_ and decreasing cerebral blood flow, the possibility arises that our observed changes in respiration parameters are not solely responsible for the observed changes in cerebral blood flow velocity. We cannot be sure whether other vasoactive substances in RB are also responsible for this novel observation, but caffeine is a likely candidate. Similarly, it is tempting to attribute the BP-elevating effects of RB to its caffeine content, but a recent pilot study [33] reported that repeated consumption of RB drinks between 8:00 and 19:00 led to an increase in mean 24 h and daytime ambulatory BP when compared to caffeine consumption alone. This raises the possibility that other ingredients in RB—in their own rights or in interaction with caffeine—may underline the BP-elevating effect of RB.

Our main findings here are that the RB drink results in an elevation in BP and diminished cerebral blood flow velocity, which contrast with the lack of effect of a similar volume of water (a control vehicle drink) on these hemodynamic parameters. There are of course numerous factors that could—via sensorial and/or metabolic effects—explain the observed differences between the RB drink and water vehicle. Further experiments are warranted to tease out the distinct component(s) of the RB drink (including sweet taste, calorie content, sugars, caffeine, taurine and glucuronolactone) that either in their own rights or through interactions with each other could be contributing to these differential hemodynamic effects.

In conclusion, our results show a negative hemodynamic profile in response to ingestion of RB in young and healthy humans and which could not be explained by impairments in endothelial function. Moreover, ingestion of an energy drink was associated with a substantial drop in cerebral blood flow, hence critically questioning the manufactures promotion about a better mental profile.
